# From skeletal muscle to stem cells: an innovative and minimally-invasive process for multiple species

**DOI:** 10.1038/s41598-017-00803-7

**Published:** 2017-04-06

**Authors:** J. Ceusters, J.-Ph. Lejeune, C. Sandersen, A. Niesten, L. Lagneaux, D. Serteyn

**Affiliations:** 1 Center for Oxygen Research and Development, Institute of Chemistry B6a, University of Liège, Sart Tilman, 4000 Liège Belgium; 2 Mont Le Soie Equine Research Center, Mont Le Soie, 1 6690 Vielsalm Belgium; 3 Department of Clinical Sciences, Equine Surgery, Faculty of Veterinary Medicine B41, University of Liège, Sart Tilman, 4000 Liège Belgium; 4 Laboratory of Clinical Cell Therapy, Institut Jules Bordet, Université Libre de Bruxelles, Campus Erasme, Brussels, Belgium

## Abstract

Bone marrow and adipose tissue represent the two most commonly exploited sources of adult mesenchymal stem cells for musculoskeletal applications. Unfortunately the sampling of bone marrow and fat tissue is invasive and does not always lead to a sufficient number of cells. The present study describes a novel sampling method based on microbiopsy of skeletal muscle in man, pigs, dogs and horses. The process includes explant of the sample, Percoll density gradient for isolation and subsequent culture of the cells. We further characterized the cells and identified their clonogenic and immunomodulatory capacities, their immune-phenotyping behavior and their capability to differentiate into chondroblasts, osteoblasts and adipocytes. In conclusion, this report describes a novel and easy-to-use technique of skeletal muscle-derived mesenchymal stem cell harvest, culture, characterization. This technique is transposable to a multitude of different animal species.

## Introduction

Adult mesenchymal stem cells are found virtually in all type of tissues^1^. They play a crucial role in homeostasis, renewal and repair of damaged tissue^2^. Bone marrow and adipose tissue are the two most commonly exploited sources of adult mesenchymal stem cells for musculoskeletal applications^3–10^. However, these sampling methods are invasive and not easily performed. This is especially true in veterinary medicine. For example, in horses, the technique of bone marrow aspirate is related to an increased risk of osteitis, osteomyelitis and even inadvertent cardiac puncture, when the puncture site is the sternum^11^. The ease of sampling, the risk of creating a lesion at the sampling site and the quantity of tissue available are important criteria when choosing the sampling technique and site. Skeletal muscles make up approximately one third of body mass, are easily accessible and should therefore be considered as the optimal source of stem cells.

Stem cells in skeletal muscle have been isolated in various species^11–16^ and by various methods, including pre-plating culture series^17–19^, repeated culture following the freeze-thaw technique^20–22^ and fluorescence activated cell sorting with cell surface makers^23^ or with Hoechst dye^24–27^. Currently, there is no standard method for the isolation of stem cells from skeletal muscles^28^.

According to the International Society for Cellular Therapy (ISCT) human cells are defined as mesenchymal stem cells when they fulfill the following criteria: the cells must be plastic-adherent, positive for some markers (CD90, CD105, CD73), negative for others (CD45, CD34, CD14, CD19 et MHC-II) and exhibit the ability to differentiate into cells of mesodermal origin such as osteoblasts, chondroblasts and adipocytes^29^. For pratical use of stem cells in regenerative medicine, they must be clearly characterized, available in sufficient quantities, harvested by minimally invasive procedures and isolated and easily cultured. As the procedures mentioned above do not meet all of these criteria, the present study proposes an alternative method for the sampling, isolation and culture of skeletal muscle-derived mesenchymal stem cells. This method is easily applied in practice and transposable to various species.

## Results

As we were initially seeking an alternative method for collecting equine pluripotent stem cells, we basically developed our technique on equine muscles. Subsequently, we have evaluated this concept on dogs, pigs and humans.

### The sampling method: muscular microbiopsy

To initiate the culture of pluripotent muscle-derived stem cells, we use muscular microbiopsies of approximately 15 to 20 mg of tissue. The sampling procedure is performed with a semi-automatic 14 gauge microbiopsy needle. The sampling site is shaved and aseptically prepared, a local anesthetic is injected subcutaneously and the microbiopsy is collected through a small skin puncture. Immediately after collection, each sample is placed in culture medium and maintained at 4 °C until use.

To date, we have sampled 45 horses, 3 pigs, 10 dogs and 2 humans with this method. All of these samples, except 3 of the dogs, were successfully cultured as described below. We neither observed any contamination of the sampling site, nor any adverse effect on muscle function except for humans who showed some painless muscle twitching for approximately 12 hours. With one microbiopsy of about 15 to 20 mg, we had sufficient tissue to easily initiate a culture. We showed also that microbiopsies can be done by veterinarians in clinical practice and do not require the hospitalisation of the animal. The absence of adverse effects and the facility of sampling enabled us to apply this technique in exercising high-performance horses. This aspect is also relevant as regards human patients.

### Initiation of the cell culture

Culture preparation was performed using sterile equipment, in the controlled environment of a biosafety cabinet. Microbiopsy specimens were washed twice in phosphate buffer saline solution (PBS), carefully dissected and then cut into small pieces. Each piece was placed individually into the 16 central wells of 24-multi-well dish pre-filled with culture medium. The multi-well dish was incubated at 37 °C in a CO_2_ incubator.

After three to four days in culture, the first cells started to appear around the muscle pieces. Depending on the species, about 10 to 18 days (13.2 days ± 2.63) after initiating the culture, a halo of cells was visible around the tissue and the number of cells was sufficient to allow for pluripotent stem cells isolation. Before the isolation step, we obtained a mean of 63000 ± 30675 cells from the 16 wells pooled together.

### Pluripotent stem cell isolation: discontinuous Percoll density gradient centrifugation

The cells emerging from explants are detached using tryspin-EDTA, centrifuged (200 × g, 10 min, 37 °C) and the pellet was suspended in Hank’s Balanced Salt Solution (HBSS). The cellular suspension was then placed on a 3 layers discontinuous Percoll density gradient (15%, 25% and 35%). The cell fractions with different densities appeared at the interfaces between each Percoll fraction after centrifugation at 1250 × g (25 °C, 20 min). To continue the culture, we chose to work with the 15–25% fraction. The fraction was washed once with HBSS and centrifuged at 200 × g, 10 min at 37 °C. The supernatant was discarded and the pellet was suspended in 1 ml of culture medium. The cells were then cultured in T-25 cm^2^ Flask.

We observed that the cells reached 80% confluence in T-25 cm^2^ Flask within 7 ± 1 days. The cells could then be frozen in liquid nitrogen or further passed for other experiments.

### Validation

The clonogenic capacities of the cells were assessed for the cells coming from each Percoll fraction (<15%, 15–25%, 25–35% and >35%) in horses (n = 3) and humans (n = 2). Their abilities to differentiate into adipocytes, chondroblasts and osteoblasts were evaluated for the cells coming from each Percoll fraction in horses (n = 5), but only for the 15–25% fraction in humans (n = 2) and dogs (n = 3). Their immunomodulatory capacities in horses (n = 2) and humans (n = 2) were also assessed. The expressions of CD90, CD105, CD44, CD45 and MHCII were studied for the cells from each Percoll fraction in 2 horses and for the 15–25% fraction in humans (n = 2) and 6 more horses. In humans (n = 2), their expression of CD34, CD19 and CD73 was also shown. Some equine bone marrow mononuclear cells were used to validate the cross-reactivity of all the antibodies used with the equine epitopes.

### Clonogenic capacities

**Figure 1:**
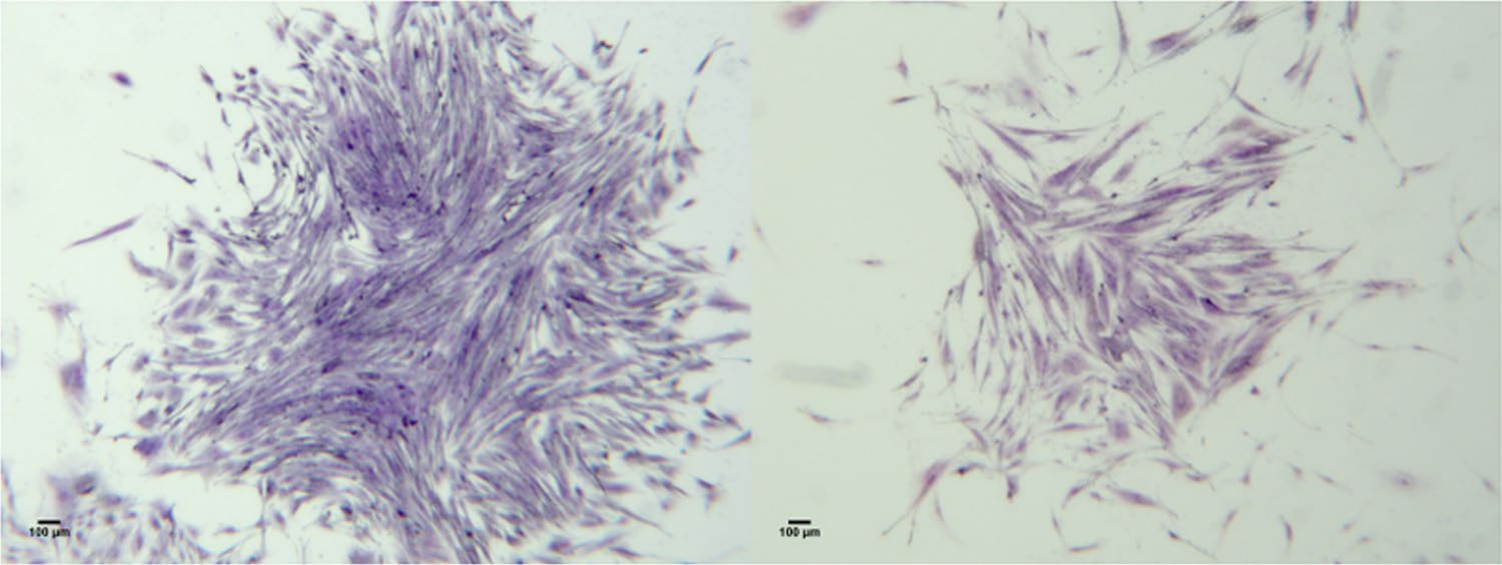
Representative microphotographies of morphological aspect of colony forming fibroblasts-like units obtained with cells from horses (left) and humans (right) (May-Grünwald Giemsa staining).

The clonogenic capacities of the cells were evaluated with a “fibroblast-colony forming units” assay (CFU-F). The morphological aspect of the CFU’s observed was not different from those habitually observed with pluripotent stem cells isolated from bone marrow or Wharton Jelly (Fig. 1). The best clonogenic capacities were observed for the 15–25% cells.

### Trilineage differentiation

**Figure 2:**
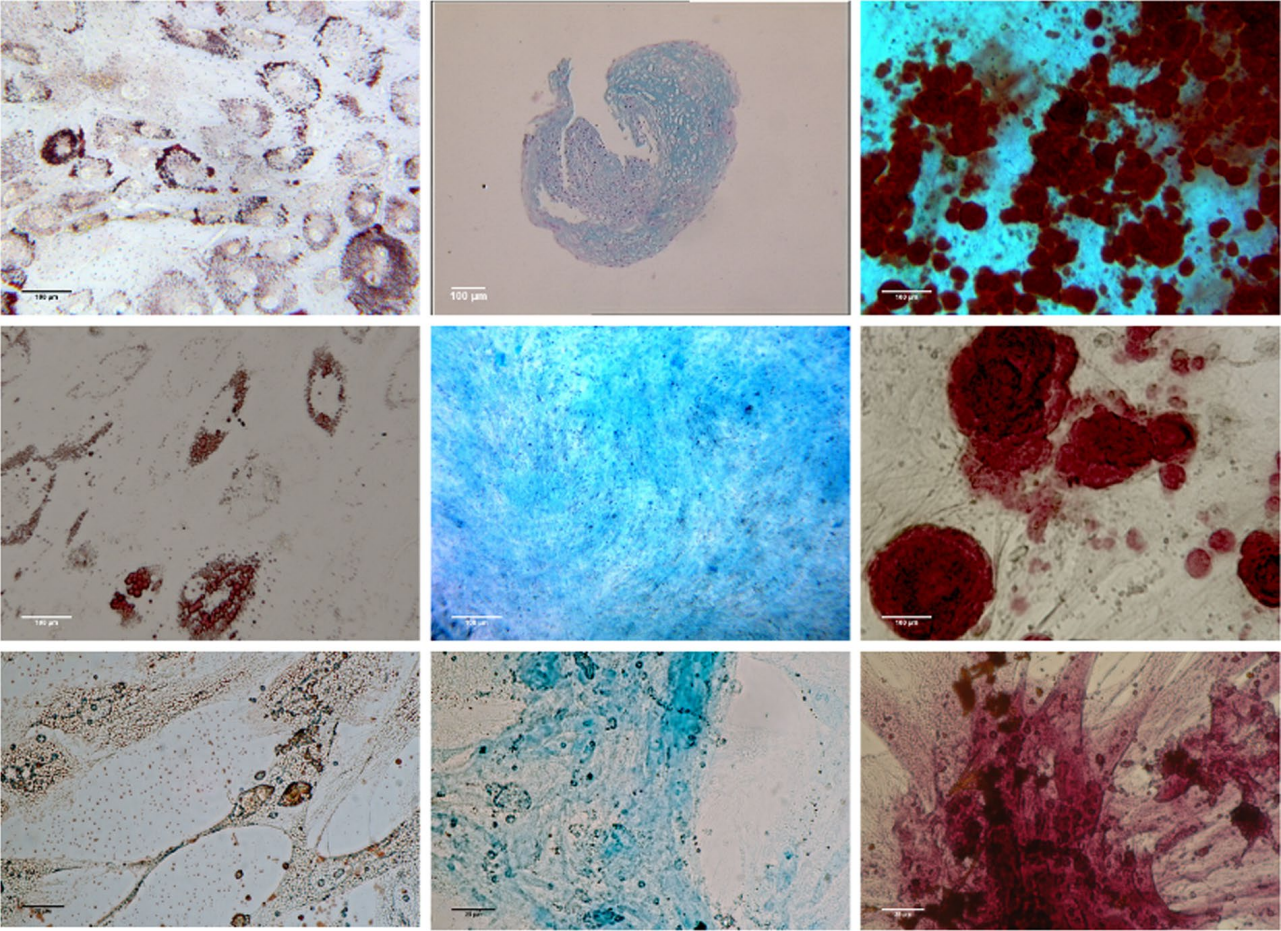
Representative microphotographies of trilineage differentiations obtained for horses (upper line), humans (middle line) and dogs (lower line). Left panel of pictures show adipogenic differentiation (Oil red O staining), middle panel of pictures show chondrogenic differentiation (Alcian bleu staining) and right panel of pictures show osteogenic differentiation (Alizarin red staining).

Adipogenic, osteogenic, and chondrogenic differentiations of equine, human and canine cells were performed in adapted specific media. The 15–25% cells obtained from each species were able to differentiate into adipocytes, chondroblasts and osteoblasts when cultured with the corresponding induction media (Fig. 2). For horses, the cells coming from each Percoll fraction were able to trilineage differentiation.

### Immunophenotyping

**Figure 3:**
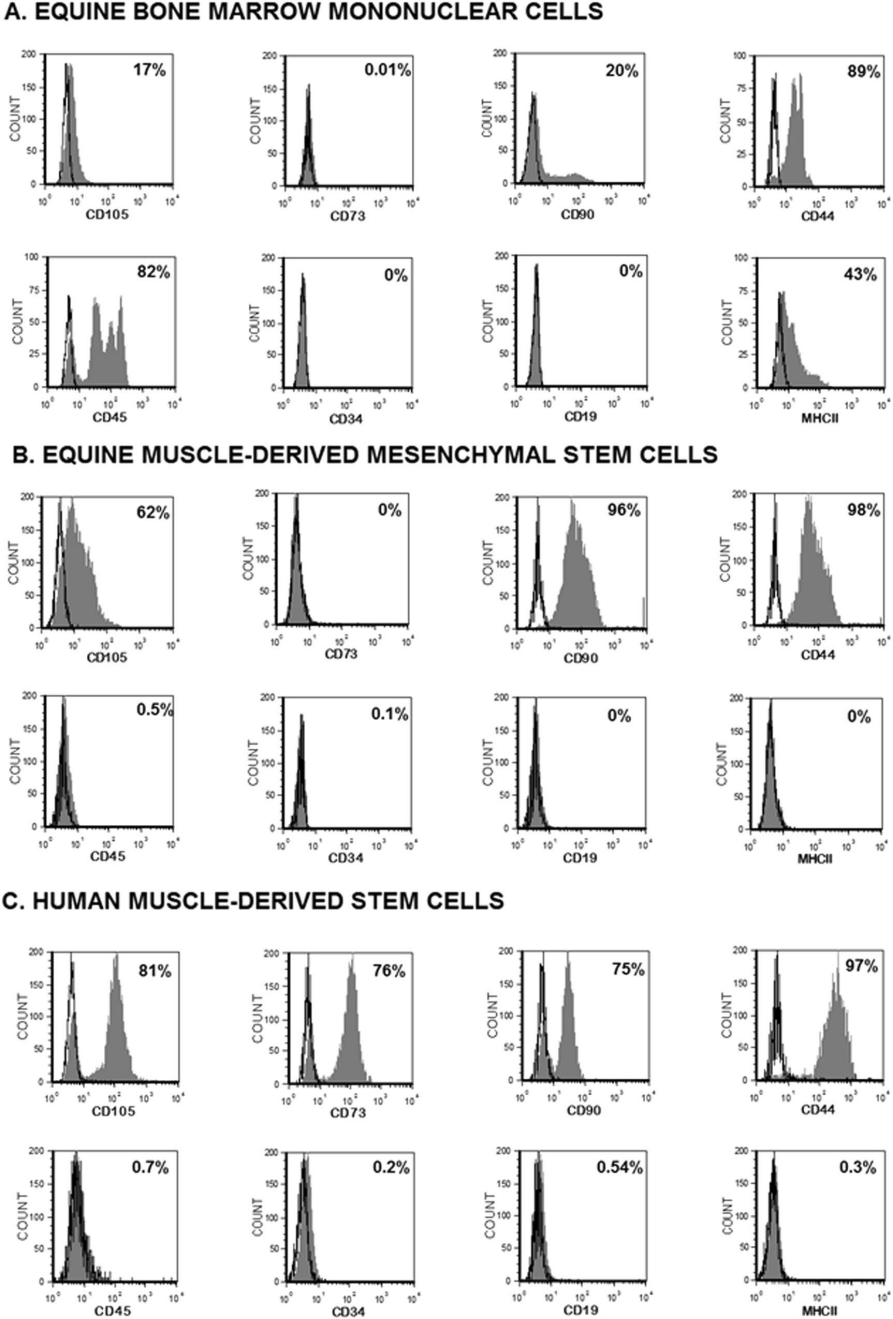
Representative flow cytometry analysis of equine and human cells: (**A**) equine bone marrow mononuclear cells (BM-MNC) (**B**) equine muscle-derived mesenchymal stem cells (**C**) human muscle-derived mesenchymal stem cells. BM-MNC were used to validate the cross-reactivity of antibodies listed in Table 1. Open histograms show nonspecific isotype control staining and solid histograms show specific staining for the indicated marker. Cross-reactivity was not found for CD73, CD34 and CD19 markers.

**Figure 4:**
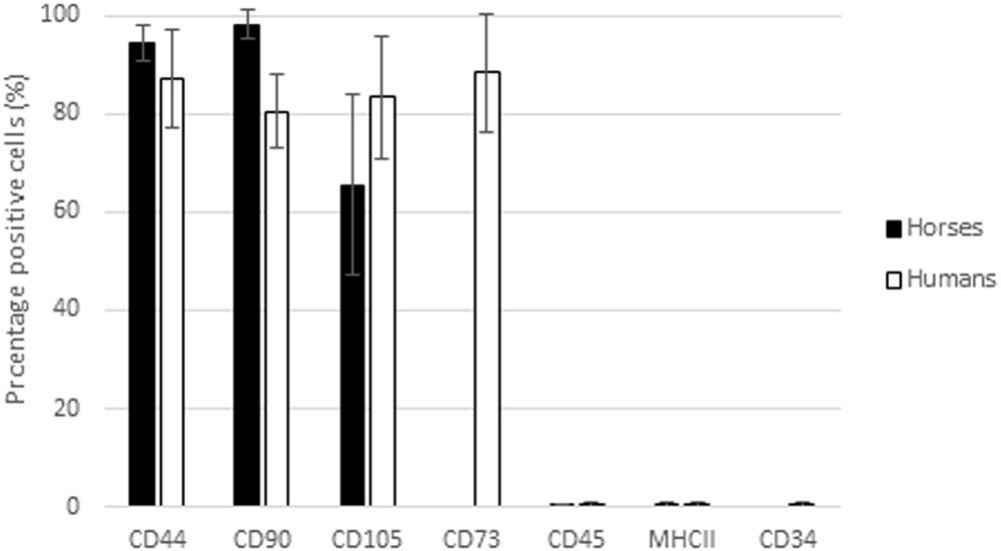
Percentage of positive cells for CD44, CD90, CD105, CD45, MHCII [human (n = 2) and equine (n = 6)], CD73 and CD34 [human (n = 2)] (Mean ± SD).

First, we validated the cross reactivity of the antibodies by using equine bone marrow mononuclear cells. As shown in Fig. 3, the CD73, CD19 and CD34 did not recognize the equine epitopes. Therefore, the equine cells were only evaluated for their expression of CD44, CD45, CD90, CD105 and MHCII. This evaluation was performed for cells from each Percoll fraction. For human cells, only those from the 15–25% fraction were used but they were further evaluated for their expression of CD73, CD19 and CD34. For the 15–25% cells from horses and humans, over 60% of the cells expressed CD105, CD44 and CD90 whereas less than 2% expressed CD45 and MHCII. Furthermore, for humans, more than 80% of cells were positive for CD73 but less than 2% expressed CD34 and CD19 (Figs 3 and 4).

For 2 horses, we characterized the cells of the different Percoll fractions. We observed that less than 2% of the cells of all fractions expressed MHCII and CD45 and more than 60% of the cells of all fractions expressed CD105 and CD44 whereas the expression of CD90 varied between the fractions. From the 2 horses tested, CD90 was expressed by 48% of the <15% cells, 73% of the 15–25% cells, 36% of the 25–35% cells and 10% of the >35% cells.

### Immunomodulatory capacities

The immunomodulatory capacities of the cells were evaluated by their ability to inhibit the proliferation of purified T-lymphocytes (LT).

An inhibition of LT proliferation (%) was observed in the presence of stem cells from the 2 species. For horses, the greatest inhibition was observed with the ratio stem cells/LT of 1/8. For human, the optimal ratio of stem cells/LT was 1/4. These patterns were similar to the ones observed with stem cells from bone marrow for the 2 species.

## Discussion

With this study, we propose for the first time an effective and patented (Mammalian muscle-derived stem cells, WO2015091210) method to obtain mesenchymal stem cells from muscles. Furthermore, this process is easily transposable to multiple species including humans.

First, the sampling method that we propose, using muscular microbiopsy is less invasive. In horses, harvesting muscle tissue is easier than bone marrow or periosteal tissue. Skeletal muscles represent about one third of body mass and the risk of inducing a lesion at the sampling site is low. However, the required quantities described by Radtke *et al*.^11^ or even by Tamaki *et al*.^28^ for their method could be prohibitive for competing horses and especially for dogs and humans. With our microbiopsy method, only 10 to 20 mg of tissue are required as against approximately 6 g according to Radtke *et al*. in horses^11^, 5 to 10 g according to Tamaki *et al*. in humans^28^, 15.3 to 42.26 g according to Kisiel *et al*. in dogs^15^ and 1 g for Lewis *et al*. in pigs^16^. A study performed by Votion *et al*.^30^ demonstrated that the microbiopsy technique can easily be performed by veterinarians in the field. Because of the ease of sampling and the absence of adverse effects, we can consider this method safe for the use in man, performance horses and other domestic animals.

Numerous authors have reported using enzymatic digestion to isolate stem cells from muscular sample^11, 13, 15, 28, 31, 32^. The quantity of tissue collected using our microbiopsy technique is insufficient for the purpose of enzymatic digestion^33^. The technique of enzymatic digestion not only requires larger quantities of tissue but also bears the risk of proteolytic contamination which can damage the cell surface ligands and receptors necessary for stem cell function after *in vivo* transplantation^28, 34^. The mechanical steps related to the method of enzymatic digestion may also be damaging for sensitive cells such as stem cells. Therefore we developed a technique that simply uses muscular microbiopsies as explants and involves waiting until progenitor cells grow from them. In this way, we reduce the number of steps involving manipulation and the potential sources of contamination. Further, sampling and cutting the biopsies to form explants mimics muscle fiber trauma and represents a trigger for stem cell activation, migration and proliferation^35–37^. The three-dimensional structure of the parent tissue during the crucial early stages of cell outgrowth is maintained while providing the outgrowing cells with a rich nutritive media in which they proliferate. Skeletal muscle explants thus mimic the *in vivo* environment of the regenerating muscle and stimulate stem cell migration and division^35^.

Muscle-derived cells are a mixture of subpopulations from different lineages and different developmental stages^38, 39^. Different techniques are used to isolate muscle-derived stem cells which can be marker profile-dependent or not. This last type of methods selects the cells on the basis of their adhesion characteristics (modified preplate technique^40^) or their densities, which are reliable in their expression of specific molecular markers^32^. Ultimately, from the perspective of clinical applications, the isolation technique must allow easy and rapid acquisition of stem cells, while respecting financial concerns in veterinary medicine. In our study, we decided to adapt a discontinuous Percoll gradient method^32^ on the cells obtained from the muscle explants. Essentially, two kinds of stem cells exist in muscle tissue: satellite cells and muscle-derived stem cells. Satellite cells, referred to by many as ‘muscle stem cells’, are ‘myogenic precursors’ capable of regenerating muscle tissue and demonstrating self-renewal properties; however, they are considered to be committed to the myogenic lineage^41^. Muscle-derived stem cells, which may represent a predecessor of the satellite cell, are considered to exhibit a higher regeneration capacity and better cell survival and a broader range of multilineage capabilities^17^. In a previous study using muscular microbiopsies as explants, our group has obtained a primary equine myoblast culture^42^. In 2002, Michal *et al*. isolated canine satellite cells with an enzymatic digestion technique^43^. These results show that the isolation step is essential to select stem cells among the muscular cell populations and suggest that applying only enzymatic digestion may be insufficient.

At first, we worked with equine samples and isolated 4 subpopulations of cells with the Percoll gradient (ie cells <15%, between 15 and 25%, between 25 and 35% and >35%). We characterized the 4 subpopulations of cells from 2 horses and observed that all of them were able to trilineage differentiation but the greatest expression of CD90 was found for the cells from the 15–25% fraction. Furthermore, the 15–25% cells showed the greatest proliferative and clonogenic capacities. Regarding these results and the data from the literature^32^ and to simplify our process, we finally choose to keep only the cells from the 15–25% Percoll fraction. This was also applied when we worked with samples from other species.

As defined by the ISCT^29^, our method allows the isolation of pluripotent mesenchymal muscle-derived stem cells from horse, human, dog and pig microbiopsies. Unfortunately, we were not allowed to use the entire panel of antibodies described by the ISCT for equine cells because the cross reactivity study showed us that CD73, CD19 and CD34 did not recognize the equine epitope. For humans, the results obtained were in accordance with the recommendations while for equine cells, only positivity for CD90, CD105 and CD44 and negativity for CD45 and MHCII was demonstrated. Even while it is known for human cells^44^, this is the first time that the expression of CD105 is described for equine skeletal muscle-derived stem cells. CD105, which is a component of the TGF beta-1 complex, has different important biological functions such as angiogenesis^45^ and growth induction at the joint/articular level. TGF beta-1 stimulates chondrocyte division as well as cartilage matrix synthesis but decreases the release of PGE2 by osteoarthritic synovial fibroblasts and hence decreases PGE2 stimulated matrix degradation in osteoarthritis^46^. CD105 expression thus appears to show excellent tissue regeneration characteristics to the obtained stem cells.

We also showed that our cells can form fibroblast-like colonies in culture, with a similar aspect to what is generally observed with other mesenchymal stem cell sources. The isolated cells can also differentiate into adipocytes, chondroblasts and osteoblasts and support a plurality of freeze-thaw cycles without losing their pluripotency. Another characteristic of the isolated cells, which is also interesting from a clinical point of view, is that they show immunomodulatory capacities towards T lymphocytes. If this is well known as regards human and equine stem cells^47–49^, it has never been shown specifically with equine skeletal muscle-derived stem cells. This anti-inflammatory property confers a real advantage in the clinical use of the cells.

To our knowledge, this is the first time such a comprehensive characterization has been described for skeletal muscle-derived stem cells from numerous species. Even if further investigations are indicated, we are now able to propose a suitable process to quickly and easily obtain fully characterized pluripotent stem cells from minimally-invasive muscular microbiopsies. These cells have distinct properties that make them particularly attractive for clinical use.

## Reagents

### General

DMEM/Ham’s F12 culture medium with Hepes and Glutamine, penicillin-streptomycin, amphotericin B, PBS, HBSS, Trypsin-EDTA from Lonza, Verviers, Belgium.

Fetal Bovine Serum (FBS), multiwell dishes from Fischer Scientific, Aalst, Belgium.

Percoll, T-flasks, conical bottom centrifuge tubes from VWR, Leuven, Belgium.

Adipogenic, osteogenic and chondrogenic differentiation media and MACSQuant Running Buffer from Miltenyi Biotec, Leiden, The Netherlands.

Alizarin Red, Alcian Blue and Oil red O from Sigma, Diegem, Belgium.

CellTraceTM CFSE cell proliferation kit from Invitrogen, Molecular Probes, OR, USA.

The semiautomatic microbiopsy needle (Temno Evolution) from Carefusion, Chateaubriant, France.

Dexmedetomidine from Orion Corporation, Finland.

Lidocaine 2% (Xylocaine) from AstraZeneca, Belgium.

### Antibodies

All information relating to the antibodies used can be found in Table 1.

**Table 1:** Antibodies for analyzing the cellular proteins on equine and human cells.

Antibody	Company	Clone	Dilution/Concentration
CD105	Abcam	SN6	1 µg/test
CD73	Abcam	10f1	1/10
CD90	VMRD	DH24A	0.5 µg/test
CD44	AbD Serotec	CVS18	1 µg/test
CD45	Serotec	F10-89-4	1/10
CD34	Miltenyi	AC136	1/10
CD19	Miltenyi	LT19	1/10
MHCII	AbD Serotec	CVS20	0.5 µg/ml

## Method

### The sampling method: muscular microbiopsy

To initiate the culture of pluripotent muscular derived stem cells, we used muscular microbiopsies which represented each approximately 15 to 20 mg of tissue. The microbiopsy procedure on animals is approved by the Animal Ethical Commission of the University of Liège and is performed in accordance with the relevant guidelines. Human samples are derived from healthy volunteer donors and informed consent is obtained. All experiments are approved by the Regional Ethical Research Committee of the Bordet Institute and conducted according to the principles expressed in the Helsinki Declaration. In this study all horses and humans were sampled without sedation, dogs were sedated with dexmedetomidine 5–10 µg/kg intramuscularly and pigs have been sampled under general anesthesia. Samples were performed by means of a 14G-sized semiautomatic microbiopsy needle in all species except for dogs for which a 16G needle was used. For horses, samples were taken from the triceps brachii muscle at a point situated halfway on a line joining the point of the elbow and the point of the shoulder. For dogs, samples were taken from the long head of the triceps brachii muscle. In pigs the samples were taken from the semitendinosus muscle. For humans, we sampled once in the biceps brachii and once in the vastus lateralis of the quadriceps femoris. For dogs and horses, the sample site is shaved and surgically scrubbed for 5 minutes. Only the surgically scrub was performed for human and pigs. A local anesthesic (lidocaine 2%) was injected subcutaneously at the sample site. After having put on the sterile gloves, a full thickness skin puncture is performed using the introducing sheath with its mandarin in place. After arming the biopsy needle (2 notches), it is slid into the introducing sheath from which the mandarin was previously removed. The biopsy needle together with the introducing sheath is inserted through the skin puncture into the muscle and advanced for 2 to 3 cm. After releasing the spring loaded mechanism, the needle with its sheath is withdrawn from the muscle. The sample is removed from the biopsy needle using a sterile disposable injection needle. The sample is carefully placed in a tube which containing DF20 medium [DMEM/Ham’s F12 culture medium with Hepes and Glutamine, 20% of heat inactivated (FBS), 5 ml penicillin (1000 U/ml)-streptomycin (10000 µg/ml) and 2.5 ml amphotericin B (250 µg/ml)]. Prior to delivery to the laboratory, the microbiopsies are kept in the DF20 at 4 °C.

### Initiation of the cell culture

Culture preparation was performed using sterile equipment, in the controlled environment of a biosafety cabinet. Two half 10 cm Petri dishes were prepared with 5 ml of PBS to wash the microbiopsy sample. A drop of PBS was placed apart in one of the half dish. The 16 central wells of a 24-multiwell dish were filled with 150 µl of DF20 and the outer wells with PBS (1 ml/well) to prevent drying out of wells containing explants. The medium containing the microbiopsy sample was poured into an empty half 10 cm Petri dish. With the help of forceps, the microbiopsy specimen was successively washed twice in the 2 different half 10 cm Petri dish containing 5 ml of PBS. Each microbiopsy specimen was carefully dissected using the sterile forceps and the scalpel blade, while attempting to retain as far as possible only muscular tissue (light pink tissue) in a half Petri dish containing 5 ml of PBS solution. The microbiopsy specimens were cut into small pieces (size of the tip of the scalpel blade) and placed apart in the drop of PBS. Each piece of muscle was individually placed into the 16 central wells of 24-mutliwell dish and incubated at 37 °C under controlled atmosphere (5% CO_2_ and 21% O_2_). The wells were monitored each day and when necessary, some DF20 was added to avoid the cells drying out (typically a single time between the initiation of culture and the isolation step).

### Pluripotent stem cells isolation

First, the discontinuous Percoll gradient was prepared. Each layer was prepared in a separate sterile 15 ml conical bottom centrifuge tube using a 9 g/l NaCl solution. For one multi-well dish containing explants, 2.5 ml of each density of Percoll (15%, 25% and 35%) were prepared by adding for 15% : 375 µl Percoll + 2.125 ml NaCl; for 25% : 625 µl Percoll + 1.875 ml NaCl and for 35% : 875 µl Percoll + 1.625 ml NaCl. Two ml of the 15% Percoll solution were placed in the bottom of a 15 ml conical bottom centrifuge tube. From below, 2 ml of the 25% Percoll solution and then 2 ml of the 35% Percoll solution were placed successively.

Within the multi-well dish, the culture medium and the pieces of muscle present in each culture well were discarded before rinsing with 500 µl of PBS. The PBS was discarded and 150 µl of trypsin added to each cultured well. The multi-well plate was placed for 2–5 minutes in the 37 °C and 5% CO_2_ incubator. When cells were detached, 300 µl of DF20 were added in each well to stop the action of trypsin. The cells were harvested from all the culture wells in a 15 ml conical bottom centrifuge tube and centrifuge for 10 min at 200 × g and 20 °C. The supernatant was removed and the cells were suspended in 1 ml of HBSS. The cellular suspension (cells in 1 ml of HBSS) was carefully placed on the Percoll gradient and centrifuge for 20 minutes at 1250 × g and 20 °C without brake. For 2 horses, each fraction (ie cells <15%, between 15 and 25%, between 25 and 35% and >35%) was harvest separately in order to characterize them for immunophenotype, clonogenic capacities and trilineage differentiation capabilities. Finally, it was chosen to work only with the cells from the 15–25% fraction so to harvest this fraction of interest, the first 2 ml were carefully removed, the following 2 ml were retained and the rest of the solution was discarded. The cellular suspension was diluted by adding 6 ml of HBSS and centrifuged for 10 min at 200 × g and 20 °C. The supernatant was discarded and the cells suspended in 1 ml of DF20. The cellular suspension was placed in a 25 cm^2^ T-Flask, 5 ml of fresh DF20 were added and the T-Flask was incubated at 37 °C in the 5% CO_2_ incubator. The medium was changed for new DF20 the day after the isolation step and again 3 days later. The isolated cells can then further be passed classically using trypsin and multiplied for up to 8 passages or cryopreserved in liquid nitrogen.

### Validation

#### Clonogenic capacities

The clonogenic capacities of the human and equine cells were evaluated with a “fibroblast-CFU” assay. Cells in primo-culture or after one passage, were seeded at low density (5000 cells/flask) and grown for 10 days. Fibroblastic colonies of more than 50 cells were scored using an inverted microscope after May Grünwald Giemsa staining.

#### Trilineage differentiation

These differentiations were carried on human, equine and canine cells.

-Adipogenic differentiation: For the adipogenic differentiation, 5000 cells/well were plated in a 24-well plate or 50000 cells/well in a 6-well plate in NH AdipoDiff Medium. After 21 days, cells were colored using Oil Red O. Briefly, cells were washed with PBS and fixed with 8% formaldehyde or 100% ethanol before staining with Oil Red O solution.

-Chondrogenic differentiation: To induce chondrogenesis, cells were transferred in the bottom of 15 ml conical tubes and differentiated into chondroblasts in pellet culture (250000 cells/pellet) in 1 ml specific chondrocyte induction medium. Tubes were incubated for 21 days at 37 °C in a 5% CO_2_ incubator, and the medium was replaced every week. Briefly, after 21 days, the micro-masses were fixed with methanol and whole mount stained with Alcian Blue.

-Osteogenic differentiation: For the osteogenic differentiation, the cells were plated in DF20 in a 24-well plate at a density of 5000 cells/well or 50000 cells/well of a 6 well multi-well dish. After 24–48 h, the osteogenic medium was added to the adherent cells. Every week, cells were fed with complete replacement of the medium. At days 21, the calcium mineralization was assessed by coloration with Alizarin Red. Briefly, cells were washed in PBS and fixed in 70% ethanol. Cells were finally stained with Alizarin Red.

#### Immunophenotyping

Human and equine muscle derived stem cells and bone marrow mononuclear cells were analyzed by flow cytometry. Briefly, the cells (10^5^) were washed with PBS and incubated with the following monoclonal antibodies (Table 1): CD105, CD44, MCH II, CD45, CD90 (equine and human cells), CD73, CD19 and CD34 (human cells only). After washing with MACSQuant Running Buffer, the cells were fixed with 4% formaldehyde solution. Data were acquired using MACSQuant Analyzer and evaluated using FCS Express 4 Flow Cytometry Software (De Novo Software, Los Angeles, CA, USA).

#### Immunomodulatory capacities

The cells obtained from human or equine muscles were plated at 4000 cells/cm^2^ in a flat-bottomed 24-well plate. After a short period of adherence, allogeneic LT purified from peripheral blood and stimulated by phyto-hemagglutinin (PHA/IL2) were incubated with the plated stem cells for 5 days of co-culture in RPMI-1640 medium supplemented with 10% FBS. We tested several stem cell:LT ratios (from 1:80 to 1:4) to investigate their importance in the stem cell mediated effects.

Lymphocyte proliferation was evaluated after carboxyfluorescein diacetate_succinimidyl ester (CFDA-SE) labeling. For CFDA-SE labeling (CellTraceTM CFSE cell proliferation kit), 10 mM CFDA-SE dye was used to stain 10^7^ LT before co-incubation with stem cells. After 5 days of co-culture CFSE fluorescence was analyzed by flow cytometry. The CFSE profile of the labeled cells was composed of several distinctive peaks representing the number of cell divisions that the proliferated lymphocytes had undergone after activation. LT proliferation was expressed by the percentage of positive LT.
